# The gatekeepers of growth: The neural roles and regulation of growth hormone‐releasing hormone neurons

**DOI:** 10.1111/jne.70117

**Published:** 2025-11-18

**Authors:** Bradley B. Jamieson

**Affiliations:** ^1^ The Francis Crick Institute London UK

**Keywords:** development, growth, growth hormone, growth hormone‐releasing hormone, somatostatin

## Abstract

The neuroendocrine control of growth is mediated by the hypothalamic–pituitary–somatic (HPS) axis. This involves the hypothalamic release of growth hormone‐releasing hormone (GHRH), which stimulates the pituitary secretion of growth hormone (GH). GH subsequently promotes growth both directly and indirectly by stimulating insulin‐like growth factor 1 (IGF1) release from the liver. While extensive research has focused on the actions and mechanisms of GH and IGF1, comparatively little attention has been given to how GHRH neurons themselves are regulated. This review aims to provide insight into how GHRH neurons are controlled, emphasizing their intrinsic electrophysiological properties and the broader brain circuitry involved in detecting physiological signals such as hormonal and metabolic status. Central to this regulation is the balance of excitatory and inhibitory inputs that generate the pulsatile secretion pattern essential for growth regulation. Somatostatin (SST) provides critical inhibitory control over both GH secretion and GHRH neuronal activity. Feedback from peripheral hormones and integration of environmental and metabolic cues can further shape GHRH neuron function. Developmental, sex‐dependent, and species‐specific variations in GHRH neuron regulation are also discussed, highlighting important avenues for future research. This review offers a neuroendocrine perspective on growth regulation, with important implications for understanding the brain's role in regulating growth and development.

## 1 INTRODUCTION

**FIGURE 1:**
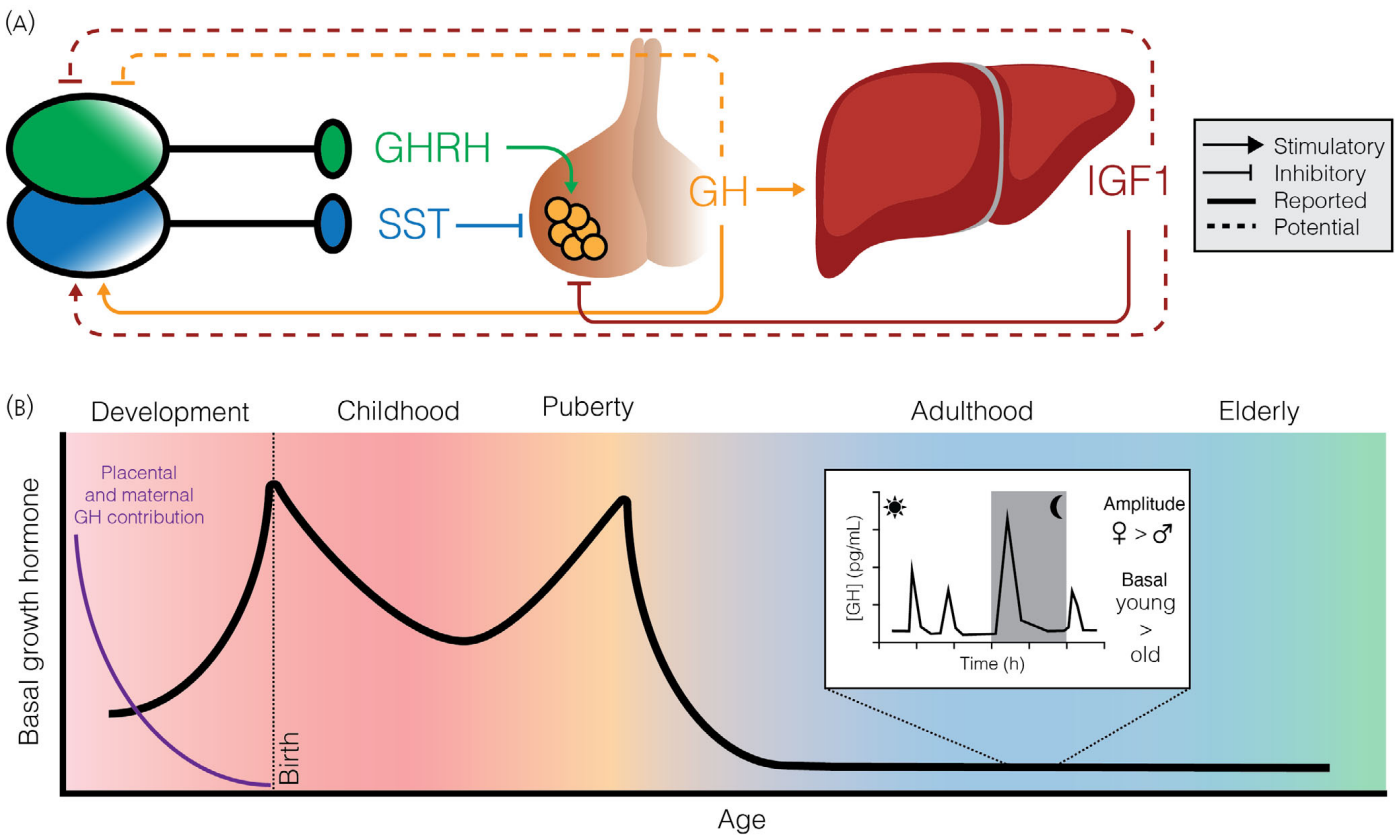
Regulation and age‐related changes in the hypothalamic–pituitary–somatic axis. (A) The hypothalamus releases growth hormone‐releasing hormone (GHRH) to stimulate growth hormone (GH) secretion from pituitary somatotrophs, which promotes growth directly or via liver‐derived insulin‐like growth factor 1 (IGF1). GH also activates somatostatin (SST) neurons to inhibit further GH release, while both GH and IGF1 provide negative feedback to the hypothalamus and pituitary. (B) GH levels change across the lifespan: Placental GH dominates in utero,^109^ followed by a postnatal rise, a dip during childhood,^27^ a peak at puberty,^110^ and a progressive decline in adulthood and aging.^29^ Inset shows pulsatile GH release across the course of a day. While females and males have similar basal GH levels, females have a typically higher GH pulse amplitude.^30^, ^31^

Vertebrate growth is intricately dependent on the body's hormonal environment, with growth factors serving as the primary positive regulators.^1^ Central to this system is growth hormone–releasing hormone (GHRH), secreted by hypothalamic neuroendocrine neurons, which controls the release of growth hormone (GH) from the anterior pituitary gland.^2^ Together, GHRH and GH orchestrate growth and development across multiple tissues through both direct and indirect actions—a system known as the hypothalamic–pituitary–somatic (HPS) axis^2^ (Figure 1A). Although physical growth ceases towards the end of puberty, GHRH neurons remain electrically active throughout life,^3^, ^4^, ^5^ suggesting roles that extend beyond growth regulation, including in sleep^6^, ^7^ and metabolism.^8^, ^9^ Importantly, GH continues to act throughout adulthood as a major regulator of metabolism, influencing energy balance and metabolic ageing.^2^, ^10^ The neural and endocrine mechanisms that sustain this lifelong GH activity are not yet fully elucidated, and while this review focuses primarily on hypothalamic GHRH neurons, these should be viewed within the broader context of GH's systemic metabolic functions. Despite the enduring activity of the HPS axis, fundamental questions remain regarding the mechanisms that regulate the electrical activity of GHRH neurons—both by central circuits and peripheral signals—and how these neurons continue to contribute to physiological functions once growth has concluded. Given these roles, dysregulation of GHRH or GH signalling has been linked to impaired somatic growth, metabolic dysfunction, and altered sleep architecture,^11^, ^12^, ^13^ highlighting that understanding GHRH neuron regulation is relevant not only for developmental biology but also for endocrine and homeostatic health across the lifespan. This review explores the regulation of the HPS axis with a focus on GHRH neurons. It highlights the electrophysiological regulation of these neurons through a dynamic balance of excitatory and inhibitory inputs that ultimately drive pulsatile GH secretion and examines the roles of negative feedback and neuromodulation.

## 2 THE HPS AXIS

GHRH was first isolated from ectopically secreting pancreatic tumours that caused acromegaly (hypersecretion of growth hormone).^14^, ^15^ Subsequent immunohistochemistry in primates identified GHRH (then known as growth hormone‐releasing factor, GRF) immunoreactive neurons in the arcuate nucleus of the hypothalamus (ARH). These GHRH neurons extend projections to the median eminence where they terminate in contact with the portal vasculature supplying the pituitary gland.^16^ It was correctly suggested that GHRH was released into the bloodstream to stimulate the release of GH.^16^ GHRH binds to a Gɑ_s_‐coupled receptor, the growth hormone‐releasing hormone receptor (GHRHR), which is predominantly located in the anterior pituitary gland somatotrophs.^17^, ^18^ Pituitary GHRHR activation results in an increase in cAMP signalling,^17^ depolarising the somatotrophs, leading to both the release of GH from secretory granules and de novo synthesis of GH and GHRHR.^19^

GH is secreted in a pulsatile manner, closely coordinated with the rhythmic release of GHRH.^20^, ^21^ The most prominent GH pulse—presumably accompanied by the highest GHRH release—occurs at the onset of sleep,^22^ a phenomenon observed across multiple species, including humans.^23^ This peak has been shown in rats to coincide with a circadian rise in *Ghrh* mRNA expression within the ARH, before the onset of the animals' sleep phase.^24^ After this initial surge, subsequent GH pulses occur approximately every 2–4 h.^20^, ^25^, ^26^ GH can be detected in the human foetal circulation as early as 10 weeks of gestation.^27^ However, studies in sheep suggest that early GH release is initially non‐pulsatile, with rhythmic secretion patterns emerging later in development.^28^ These findings suggest that, at least in sheep, basal pituitary GH secretion may be established prior to hypothalamic regulation of the HPS axis. By the time of birth, GH secretion is clearly pulsatile and present at concentrations significantly higher than those observed in childhood or adulthood. Following puberty, GH levels decline markedly and remain low throughout adult life.^29^ Interestingly, while basal serum GH levels are comparable between adult males and females, women typically exhibit higher GH pulse amplitudes.^30^, ^31^ This sex difference appears to be mediated by oestrogen, as pulse amplitude rises across the menstrual cycle in parallel with increasing oestrogen levels^30^ (Figure 1B). Supporting this, GHRH‐specific knockout of oestrogen receptor alpha leads to growth reduction in both male and female mice, indicating a direct role for oestrogen in promoting GH output.^32^ Consistently, men treated with oestrogen develop a more female‐like GH pulsatility profile.^30^

With ageing, additional changes emerge within the HPS axis. Data from rats suggests that up to a quarter of ARH GHRH neurons are lost,^33^ accompanied by a corresponding decrease in pituitary GHRHR expression,^34^ which may underlie the decline in GH secretion. Whether this reflects reduced receptor availability driving lower secretion, or a secondary consequence of diminished GHRH release from the smaller neuronal pool, remains unclear.^35^ Intriguingly, this loss of GHRH neurons occurs in parallel with an increase in arcuate kisspeptin (Kiss1) neurons after puberty, raising the possibility of neuronal phenotype switching.^32^ Notably, Kiss1 can induce GH release in several species,^36^, ^37^, ^38^ potentially from direct actions at the pituitary. Kiss1 neurons, however, may indirectly modulate the somatotropic axis, as discussed in Section 4.3, linking reproductive, metabolic, and growth pathways.

Once in the periphery, GH acts as an anabolic hormone via the GH receptor (GHR). The GHR is a type 1 cytokine receptor^39^ that can either directly stimulate tissue growth, or act at the liver to produce and stimulate the release of insulin‐like growth factors (IGFs). IGFs, particularly IGF1, work in conjunction with GH to promote tissue growth through action at their own receptor, IGF1R, and the insulin receptor.^40^ The peripheral actions of GH and IGF1 have been reviewed extensively elsewhere.^1^, ^41^, ^42^

Initially in situ hybridisation studies in the rat brain suggested that only ~10% of arcuate GHRH neurons express c‐Fos in response to GH administration,^43^ indicating a limited transcriptional response to GH in these cells. Given that distinct signalling pathways can be activated by receptor binding, more recent work in mice has shown that ~60% of GHRH neurons exhibit pSTAT5 activation following systemic GH injection,^44^ while IGF1 bolus administration induces pAKT expression in the majority of these neurons.^45^ This apparent discrepancy may reflect differences between transcriptional vs. signalling readouts, but in any case, suggests that sustained GH/IGF1 signalling can act directly on GHRH neurons, even if acute activity effects are limited. More broadly, pSTAT5 mapping reveals widespread GH‐response regions across the mouse brain,^46^ implying that indirect central feedback—from these GH‐sensitive nodes to GHRH or SST neurons—could either facilitate or inhibit GH release.

Negative feedback within the HPS axis is further complicated by parallel actions of GH and IGF1 on pituitary somatotrophs as well as on hypothalamic circuits.^47^, ^48^ In practice, the axis is predominantly self‐regulated by somatostatin (SST) neurons, which provide strong inhibitory control over both GHRH neuron activity and GH secretion. SST neurons are present within the ARH and surrounding ventro‐ and dorsomedial hypothalamic nuclei (VMH, DMH), as well as more anterior hypothalamic regions including the para‐ and periventricular nuclei (PVN, PeN), and median preoptic area (MPOA). Only the anterior SST neuronal clusters are hypophysiotropic, and release SST into the pituitary portal system.^6^, ^49^, ^50^ SST acts through 5 receptors (SSTR1‐5), all of which are present in the pituitary gland (although the expression of SSTR4 is comparatively negligible).^51^ SST hyperpolarises somatotrophs thereby inhibiting GH secretion.^52^ Posterior hypothalamic SST clusters in and around the ARH, while not hypophysiotropic, suppress the activity of GHRH neurons, as discussed below.

Our understanding of the HPS axis mostly comes from issues that arise with mutations in parts of the axis itself. Mutations in the *Ghrh* gene itself are not commonly reported in humans,^53^ although GHRH is successfully used as treatment for idiopathic GH deficiency (IGHD),^54^ suggesting an initial disruption in GHRH release or action. GHRH‐KO mouse models display a similar lack of linear growth, with very low levels of serum GH and IGF1.^13^ Mutations in the *Ghrhr* gene are more characteristic of IGHD. A single point mutation in *Ghrhr* prevents GHRH binding, and causes not only decreased GH/IGF1 levels, but also impaired pituitary development.^55^, ^56^ This mutation has since been harnessed in the ‘little’ (*Ghrhr* ^*lit/lit*^) mouse line, widely used as a disease model for IGHD.

## 3 GHRH NEURONS

GHRH‐expressing neurons in the ARH are essential for initiating the HPS axis and supporting the development of pituitary somatotrophs.^55^, ^56^ Beyond this canonical population, in situ hybridisation studies in the mouse brain reveal a broader distribution of *Ghrh*‐expressing neurons extending along the ventricular border of the VMH and DMH, with widespread expansion throughout the zona incerta (ZI).^57^ These neurons emerge during mid–late foetal development^58^, ^59^ and are induced by the transcription factor distal‐less homeobox 1 (*Dlx1*/DLX1). *Ghrh*‐expressing neurons are initially concentrated in the ARH, spreading laterally and dorsally to populate the edges of the VMH and DMH.^60^

In the embryonic ARH, *Dlx1* expression demarcates GHRH neurons from other hypothalamic populations, with no overlap observed between *Dlx1* and other key ARH peptides such as Kiss1, agouti‐related peptide (AgRP), neuropeptide Y (NPY) or pro‐opiomelanocortin (POMC).^58^ DLX1 also suppresses the expression of orthopedia homeobox (*Otp*), a gene essential for the development of AgRP/NPY neurons, indicating that GHRH and canonical metabolic neurons are specified by distinct developmental programs. As a result, ARH GHRH neurons rarely co‐express metabolic peptides and show minimal colocalisation with AgRP/NPY or POMC.^8^, ^61^ Interestingly, this may not be the case in human GHRH neurons which show co‐expression with AgRP.^62^ GHRH neurons are predominantly GABAergic, although subsets express a diverse array of neuropeptides and neurotransmitters—including galanin (Gal), neurotensin, acetylcholine (ACh), and dopamine (DA)^44^, ^63^, ^64^—as well as exhibiting an age‐dependent overlap with Kiss1 expression, reflecting the reported GHRH‐Kiss1 phenotypic shift around puberty.^32^ None of these markers, however, show exclusive overlap with GHRH. Notably, co‐expression of tyrosine hydroxylase, a marker of DA synthesis, appears to be sexually dimorphic, with more dopaminergic GHRH neurons observed in females,^64^ though the functional relevance of this remains unclear.

A minor population of *Ghrh*‐expressing neurons also appears developmentally in the rodent ventral pallidum (VPa), later contributing sparse populations to the MPOA and PVN.^60^, ^65^ These extra‐arcuate GHRH neurons are primarily glutamatergic and frequently co‐express Gal and thyrotropin‐releasing hormone (TRH).^66^ Their functions remain unknown.

Advances in genetic tools have allowed for more precise mapping of GHRH expression. The *GHRH‐eGFP* mouse line couples enhanced green fluorescent protein (eGFP) expression with active GHRH production, revealing nearly complete colocalisation with GHRH in the ARH, with some additional dorsal expression.^3^ A complementary reporter model, the *GHRH‐Cre:eGFP‐L10* mouse, expresses *eGFP‐L10* upon the first occurrence of GHRH expression,^67^ enabling the tracing of transient developmental expression. Interestingly, this model reveals putative developmental GHRH expression in the periaqueductal grey and nucleus tractus solitarius,^67^ regions not typically associated with GHRH expression in the adult brain.^57^, ^65^

Species differences further complicate the study of GHRH neurons: the human GHRH peptide sequence differs by ~40% from that of rodents,^68^ limiting the utility of typically human‐based antibodies in rodent tissue. Consequently, RNA‐based in situ hybridisation remains the most reliable method for identifying GHRH‐expressing cells in rodents. In contrast to rodents, immunohistochemical studies in the human brain reveal a much broader distribution of GHRH neurons, with cell bodies extending from the lateral and posterior hypothalamus through the infundibular (human ARH) nucleus to the borders of the mammillary bodies.^63^

While the primary projection target of GHRH neurons is the median eminence, this does not preclude additional central projections. Indeed, GHRH‐immunoreactive fibres have been identified throughout the hypothalamus, including the MPOA, PVN, and PeN, as well as in extrahypothalamic regions such as the bed nucleus of the stria terminalis (BNST), nucleus of the diagonal band (NDB), and medial amygdala (MeA).^65^ Although the specific origin of these projections remains unresolved, evidence suggests that only the ARH GHRH neurons project to the median eminence and are thus considered hypophysiotropic.^69^ Notably, ARH GHRH fibres have also been observed forming close appositions with GHRH neuron somata,^70^ hinting at potential autoregulatory feedback or mechanisms for synchronisation within the population.

## 4 ELECTRICAL REGULATION OF GHRH NEURONS

GHRH neurons exhibit spontaneous firing activity at the first postnatal week (and likely earlier, given the pattern of embryonic GH secretion) in ex vivo slice preparations, with baseline firing frequencies comparable to those observed in adulthood.^71^ However, their excitability is significantly heightened during early development. In response to depolarizing current, GHRH neurons from pre‐weaning mice fire more action potentials, show shorter latencies to firing, and exhibit broader action potential half‐widths compared with adults,^71^ possibly underlying changes in GHRH secretion. These developmental differences likely reflect the maturation of intrinsic membrane properties, particularly a potassium current that emerges post‐weaning and contributes to delayed firing in mature neurons.^71^ Notably, the age‐related increase in action potential half‐width appears absent in gonadectomised juvenile mice,^71^ consistent with modulation by pubertal hormonal changes. These age‐dependent changes in excitability may underlie the altered patterns of GH secretion observed between juveniles and adults.

In adulthood, murine GHRH neurons display limited intrinsic oscillatory activity,^72^ as shown in ex vivo recordings. However, they are subject to dynamic synaptic remodelling that correlates with the pulsatile GH secretion pattern described above. Glutamatergic (VGLUT2‐positive) synapses on GHRH neurons are approximately twofold higher during GH peaks than during troughs.^73^ In parallel, there is an increase in GABAergic (VGaT‐positive) synapses and the SST receptor SSTR1 is more prominently expressed on the GHRH neuronal membrane during GH troughs,^73^, ^74^ suggesting heightened inhibitory tone during these periods. Together, these observations point to a complex interplay of excitatory and inhibitory inputs that coordinate the pulsatile release of GH via GHRH neuron activity.

### 4.1 Negative regulation of GHRH neuron activity

Electrophysiological recordings in mice expressing fluorophores linked to GHRH production have confirmed that GHRH neurons receive direct synaptic inputs mediated by glutamate and GABA.^3^, ^71^, ^72^ However, much of our understanding of GHRH neuron regulation has been inferred indirectly from patterns of GH secretion, which complicates interpretation. A list of studied regulators likely having direct effects on GHRH neurons is outlined in Table 1. Pharmacological interventions, for example, may act directly on the pituitary or indirectly via central circuits that influence GHRH neuron activity. Hypothalamic explant studies have been used to assess GHRH release into the surrounding media as a proxy for neuronal activation,^75^, ^76^ yet these approaches cannot always exclude upstream network involvement.

**TABLE 1:** Neural changes affecting GH release in response to different stimuli.

Regulator	Technique	Tissue	Age/species/sex	Result	References
GHRH	Patch‐clamp electrophysiology	Acute hypothalamic slices with identified GHRH neurons	10–16 w/Mouse/MF (*Ghrh‐eGFP*)	No change in GHRH firing rate	[5]
GH	Patch‐clamp electrophysiology	Acute hypothalamic slices with identified GHRH neurons	8–12 w/Mouse/MF (*Ghrh‐cre:eGFP‐L10*)	No acute change in GHRH membrane potential or firing rate	[44]
	Immunohistochemistry	Fixed hypothalamic slices	8–12 w/Mouse/MF (*Ghrh‐cre:eGFP‐L10*)	Increased pSTAT5 expression in GHRH neurons	[44]
	Single systemic GH injection followed by Northern blot	ARH tissue block	8–10 w/Rat/MF (Dwarf rat)	Decreases *Ghrh* mRNA	[111]
	7‐day systemic infusion of GH followed by Northern blot	ARH tissue block	8–10 w/Rat/MF (Dwarf rat)	Decreases *Ghrh* mRNA	[111]
IGF1	Patch‐clamp electrophysiology	Acute hypothalamic slices with identified GHRH neurons	8–12 w/Mouse/MF (*Ghrh‐cre:eGFP‐L10*)	No acute change in GHRH membrane potential or firing rate	[44]
	Single systemic IGF1 injection followed by Northern blot	ARH tissue block	8–10 w/Rat/MF (Dwarf rat)	No change in *Ghrh* mRNA	[111]
	7‐day systemic infusion of IGF1 followed by Northern blot	ARH tissue block	8–10 w/Rat/MF (Dwarf rat)	Decreases *Ghrh* mRNA	[111]
SST	Patch‐clamp electrophysiology	Acute hypothalamic slices with identified GHRH neurons	12–16 w/Mouse/MF (*Ghrh‐eGFP*)	Decrease in GHRH neurons firing rate and membrane potential via SSTR1/2 activation of GIRK channels.	[4]
				Dampens glutamatergic input (♀) and/or GABAergic input (♂)	[4]
	SST injection to mediobasal hypothalamus	Repeated blood sample	Adult/Rat/M (Porton or Sprague–Dawley)	Nanomolar SST causes increases in GH output	[78]
NPY	ICV administration	Repeated blood sample	Adult/Rat/M (Wistar)	Decreases GH output via SST	[82]
	Patch‐clamp electrophysiology	Acute hypothalamic slices with identified GHRH neurons	12–16 w/Mouse/MF (*Ghrh‐eGFP*)	Increases GHRH firing	[4]
DA	In vitro incubation and radioimmunoassay	Median eminence fragment	Adult/Rat/M (Sprague–Dawley)	Likely stimulates GHRH release (see below)	[75]
Gal	In vitro incubation and radioimmunoassay	Median eminence fragment	Adult/Rat/M (Sprague–Dawley)	Stimulates GHRH release via DA receptor action	[75]
NAd	Systemic administration	Repeated blood sample	Adult/Rat/M (Wistar)	Stimulation of GHRH release by ɑ2 agonist	[87]
	Systemic administration	Repeated blood sample	Adult/Rat/M (Sprague–Dawley)	Inhibition of GHRH release by β2 agonist	[88]
ACh	Anticholinesterase administration to increase ACh content	Repeated blood sample	9–11 m/Sheep/M (Merino)	Increase in GHRH output	[90]
5‐HT	ICV administration	Repeated blood sample	Adult/Rat/M (Sprague–Dawley)	Increase in GH output	[91]
POMC	β‐Endorphin intravenous	Single blood sample	44–46 d/Rat/M (Sprague–Dawley)	Increases GH (but not without hypothalamus)	[94]
Ghrelin	Patch‐clamp electrophysiology	Acute hypothalamic slices with identified GHRH neurons	10–16 w/Mouse/MF^5^ 8–13 w/Mouse/M^98^ (*Ghrh‐eGFP*)	Increases GHRH firing rates	[5, 98]
				No change to GABA and glutamate postsynaptic currents	[5]
				Decreases GABAergic (but not glutamatergic) postsynaptic currents	[98]
Leptin	ICV administration followed by Northern Blot	Whole hypothalamic tissue block	8 m/Rat/M (Sprague–Dawley)	Increases *Ghrh* mRNA	[99]
	ICV administration	Repeated blood sample	Adult/Rat/M (Wistar)	Increases GH pulse amplitude	[100]
	Weight and mass measurements	Whole mouse	Adult/Mouse/MF (LepRb^Ghrh^KO)	No changes in body composition	[67]
Kiss1	Immunohistochemistry	Fixed hypothalamic slices	Adult/Sheep/F (Suffolk‐mix)	Increased c‐Fos in GHRH neurons; decreased c‐Fos in SST neurons	[38]
Glucose	2‐Deoxyglucose administration followed by immunohistochemistry	Fixed hypothalamic slices	12–30 w/Mouse/MF (*Ghrh‐eGFP*)	Increased c‐Fos in GHRH neurons	[8]
	Patch‐clamp electrophysiology	Acute hypothalamic slices with identified GHRH neurons	3–8 w/Mouse/M (*Ghrh‐eGFP*)	Lowered glucose levels increase GHRH firing rate and membrane potential	[9]
	Cultured explant	Acute hypothalamic slice	Adult/Rat/M (Wistar)	Increasing glucose in medium inhibits GHRH secretion	[76]

*Note*: Ages are given in months (m), weeks (w) or days (d) where reported, or where reported as ‘adult’. Animal breed/strain is given in parentheses below. Sex reported as male (M), female (F) or both (MF).

A major modulator of GHRH neuron activity is SST, which inhibits both pituitary GH secretion and GHRH neuronal excitability. SST application silences GHRH neurons via SSTR1 and SSTR2 receptors, primarily through activation of an inwardly rectifying potassium current that hyperpolarises the membrane and reduces action potential firing.^4^ In addition to the postsynaptic action, SST modulates presynaptic transmission onto GHRH neurons suppressing both excitatory and inhibitory synaptic input in a sex‐dependent manner: glutamatergic input is reduced in females, whereas GABAergic input is reduced in males.^4^ The functional significance of this dimorphism for GH pulse generation remains uncertain. Notably, the magnitude and direction of SST's effects may vary across hormonal states, and could also occur indirectly via sexually dimorphic co‐expression of other modulators such as DA^64^ (see Section 3).

**FIGURE 2:**
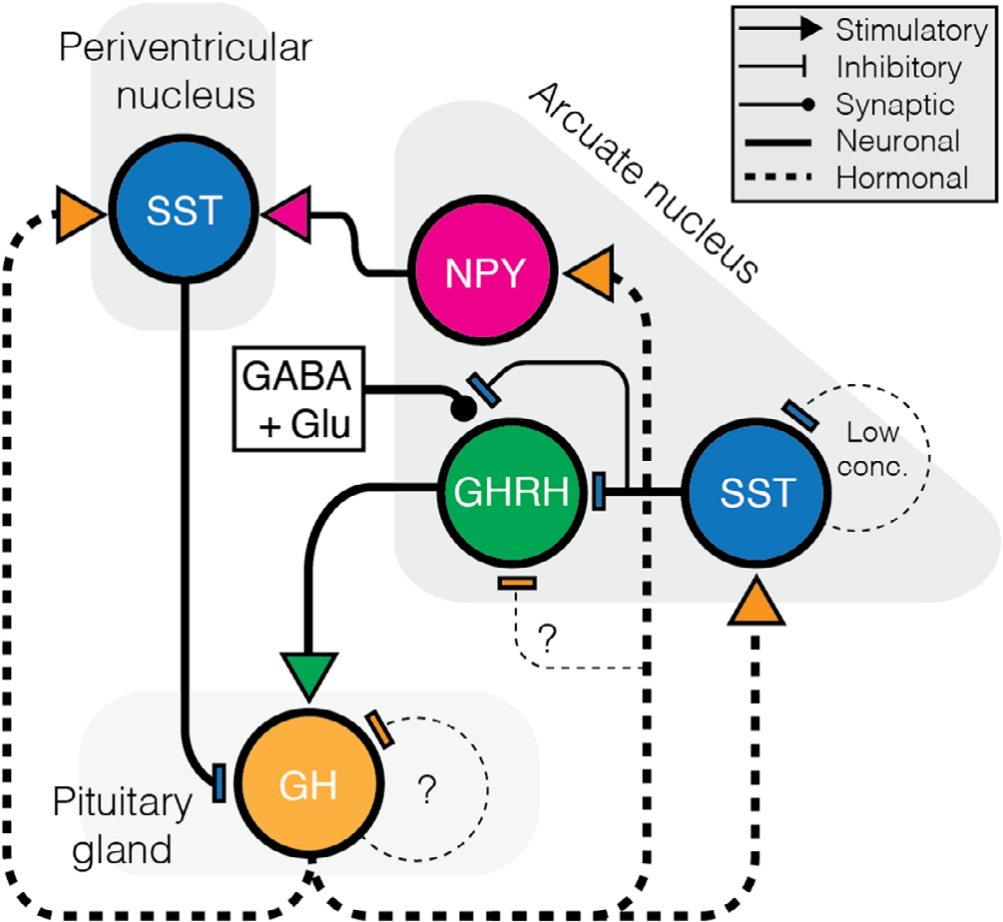
Hypothesised neural feedback regulation of GH secretion. GHRH neurons stimulate GH release from the pituitary. GH feeds back through multiple pathways: (1) activating SST neurons in the periventricular nucleus to inhibit GH release; (2) acting via arcuate nucleus NPY neurons to promote SST‐mediated inhibition; and (3) suppressing GHRH neuron activity and reduced synaptic input through arcuate SST neurons. The role of GH in direct autoregulation and long‐term modulation of GHRH neurons remains unclear.

Anatomically, hypophysiotropic SST neurons (from the PeN) rarely project to GHRH neurons,^6^, ^77^ suggesting that SST‐mediated inhibition originates largely within the arcuate nucleus itself. One proposed model is that GH release, stimulated by GHRH at the pituitary, feeds back to inhibit its own production through multiple pathways: by activating PeN SST neurons and by recruiting arcuate SST neurons that suppress GHRH neurons locally. In this framework, PeN SST neurons primarily mediate pituitary‐level inhibition of GH secretion, whereas ARH SST neurons provide local inhibitory control over GHRH neuronal excitability (Figure 2). This dual feedback architecture may help drive the pulsatile nature of GH secretion. Curiously, SST appears to exhibit autoinhibitory properties at low concentrations through SSTR1,^78^, ^79^ which may paradoxically enhance GH secretion by lifting the inhibitory tone on both GHRH neurons and the pituitary.

While SST‐mediated feedback is well studied, potential autoregulation of GHRH neurons by GHRH itself remains largely unexplored. Meanwhile, GH feedback likely acts at the hypothalamic level, given the widespread expression of GH receptors in both the preoptic and arcuate nuclei.^80^ GH has been shown to activate immediate early gene expression in PeN SST neurons,^81^ consistent with a direct negative feedback mechanism. Rather than acutely suppressing GHRH neurons directly,^44^ GH appears to act indirectly through arcuate NPY/AgRP neurons.^81^, ^82^ These neurons are directly depolarised by GH, exhibit pSTAT5 induction and upregulate both *Npy* and *Agrp* transcripts.^83^ This action likely contributes to an NPY → SST → pituitary inhibitory circuit,^82^ reinforcing GH feedback. In addition, NPY can depolarise GHRH neurons,^5^ suggesting that GH activation of NPY/AgRP may also enhance GHRH excitability over longer timescales, as observed in food‐restricted mice where GH levels are elevated.^83^ Similarly, IGF1, a downstream effector of GH, promotes SST expression while suppressing GHRH,^79^ although whether these effects are direct or circuit‐mediated remains to be clarified.

Together, these findings highlight that negative regulation of GHRH neurons is orchestrated through a multilayered network: direct SST inhibition at both pituitary and hypothalamic levels, indirect feedback via GH‐ and IGF1‐sensitive circuits, and possible autoregulatory mechanisms that remain to be explored.

### 4.2 Neuromodulation of GHRH neurons

Arcuate GHRH neurons co‐express DA and Gal^64^ (and potentially Kiss1,^32^ as mentioned above), and are also innervated by dopaminergic fibres, as evidenced by tyrosine hydroxylase (TH)‐positive appositions.^84^ The role of DA in GHRH regulation is complex, as it exerts effects both centrally and peripherally. At the pituitary level, DA acting via D1 receptors promotes GH release,^85^ whereas within the hypothalamus, modulation may occur through D2 receptors (D2Rs) expressed on GHRH neurons.^75^ Gal appears to facilitate GHRH release indirectly via dopaminergic pathways.^75^ Supporting this, mice lacking D2Rs exhibit marked growth impairments and pituitary hypoplasia^86^—phenotypes consistent with disrupted GHRH signalling—although these effects likely reflect broader pituitary dysfunction, and cannot be attributed solely to loss of hypothalamic dopaminergic modulation.

Further complexity arises from the potential role of adrenergic signalling. DA can be enzymatically converted to noradrenaline (NAd) and adrenaline by dopamine‐β‐hydroxylase (DBH) and phenylethanolamine‐N‐methyltransferase (PNMT), respectively. Pharmacological studies in male rats suggest that α2‐adrenergic receptor agonists enhance GHRH release,^87^ while β2‐adrenergic receptor agonists inhibit it.^88^ However, DBH‐ and PNMT‐expressing fibres are not observed in proximity to GHRH neurons within the human ARH^84^ implying species‐different effects or that adrenergic effects may be mediated indirectly. The relative influence of dopaminergic and adrenergic signalling on GHRH neurons remains unresolved.

**FIGURE 3:**
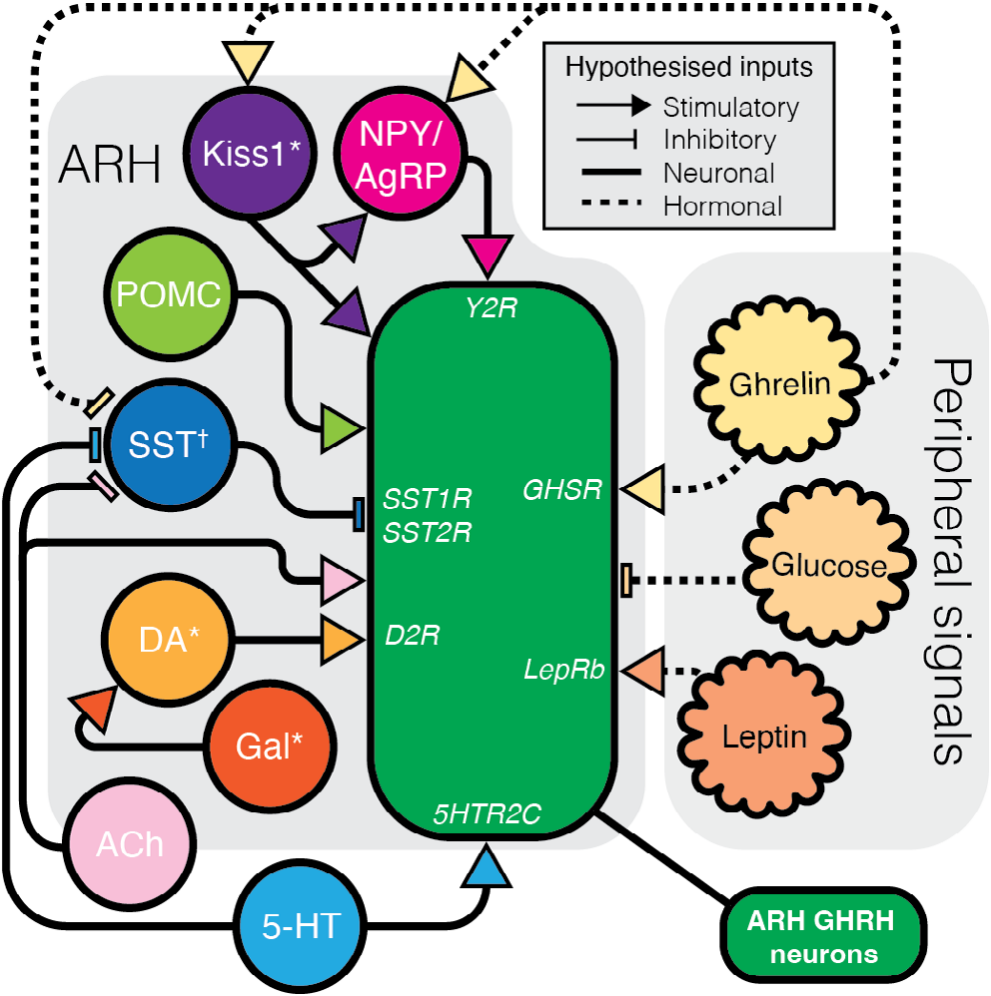
Hypothesised neural modulation of GHRH neurons. A hypothesised schematic of inputs and circuits to GHRH neurons that can modulate GHRH activity or GH output, without conflicting actions at the pituitary. NPY,^4^ Kiss1,^38^ β‐endorphin,^94^ ACh,^90^ DA,^75^ 5‐HT,^91^ leptin^100^ and ghrelin^5^, ^98^ all provide stimulatory inputs, while SST,^4^ and glucose^9^ are inhibitory. Receptors that have been implicated in GHRH modulation are also noted. * indicates neurons that can co‐express GHRH. ^†^ indicates that SST neurons could also be affected in the PeN rather than solely the ARH population.

Cholinergic and serotonergic inputs also play important roles. Increased ACh in sheep enhances GH output by suppressing SST tone, thereby disinhibiting GHRH neurons,^89^ as well as possibly stimulating GHRH neurons directly.^90^ Similarly, serotonin (5‐HT) promotes GH release through hypothalamic mechanisms, potentially by decreasing SST‐mediated inhibition of GHRH neurons or by directly activating them.^91^ These effects are likely mediated by 5‐HTR1D and 5‐HTR2C receptors respectively,^92^ although most evidence comes from systemic or intracerebroventricular administration rather than circuit‐mapped inputs. Together, these findings suggest that GHRH neurons are subject to diverse neuromodulatory influences (Figure 3) which likely act in concert with SST‐mediated inhibition to fine‐tune growth hormone output.

### 4.3 Metabolic regulation of GHRH neurons

Within the ARH, GHRH neurons are embedded among metabolically relevant populations, including NPY and POMC neurons. NPY has been shown to enhance GHRH neuronal activity through the Y2 receptor in mice,^5^ potentially contributing to increased GH release. Similarly, POMC‐derived peptides—namely α‐melanocyte‐stimulating hormone and β‐endorphin—can elevate GH secretion in rats,^93^, ^94^ potentially through direct activation of GHRH neurons^87^ or indirectly by attenuating somatostatinergic inhibition of the pituitary.^95^ While these interactions suggest an integration of growth and metabolic signalling, the precise mechanisms remain poorly defined. Conversely, negative energy balance can also suppress GH output, largely through enhanced SST tone,^96^ highlighting that metabolic state can bidirectionally regulate the HPS axis.

Ghrelin, a well‐characterised metabolic hormone, is among the most potent activators of GHRH neurons. Acting via the growth hormone secretagogue receptor (GHSR), which is expressed in both the ARH and the pituitary,^97^ ghrelin appears to exert a dual action—directly exciting GHRH neurons and stimulating GH release at the pituitary. GHSR activation increases the firing rate of GHRH neurons and simultaneously reduces inhibitory GABAergic input,^5^, ^98^ thereby disinhibiting these cells and promoting GH output. Interestingly, ~45% of GHRH neurons also express the leptin receptor (LepR).^67^ Although leptin and ghrelin often act in opposition in the context of energy balance, both converge on GHRH neurons to promote GH secretion. Leptin administration increases *Ghrh* mRNA expression^99^ and amplifies GH pulse amplitude (without altering frequency) in rats,^100^ suggesting a potential role in replenishing intracellular GHRH stores to facilitate subsequent ghrelin‐induced release. Despite this, mouse models of LepR knockout in GHRH neurons do not display significant alterations in body composition,^67^ indicating that leptin's influence on GH dynamics may be more nuanced and context‐dependent.

Beyond the direct effects on GHRH neurons, ghrelin can also act at arcuate Kiss1 neurons, which can, in turn, influence GHRH‐associated networks. In mice, arcuate Kiss1 neurons express GHSR, whose expression is up‐regulated by oestradiol.^101^ Ghrelin also depolarises a subset of these neurons.^101^ This indicates that during negative energy balance, when ghrelin levels are elevated, the concurrent presence of oestrogen could enhance Kiss1 neuron excitability. In sheep, central administration of Kiss1 induces c‐Fos expression in GHRH and NPY neurons while reducing activity in SST neurons,^38^ suggesting a hypothalamic mechanism through which Kiss1 neurons may facilitate GH release indirectly. Together, these findings support a model in which ghrelin‐responsive Kiss1 neurons recruit NPY and GHRH populations to form a metabolic–reproductive relay (Kiss1 → NPY → GHRH) that coordinates GH secretion with nutritional and hormonal state.

## 5 EMERGING ROLES OF GHRH OUTSIDE GROWTH

Beyond their well‐established role in regulating GH secretion, GHRH neurons are gaining recognition for their involvement in a range of other physiological processes. Recent research highlights key functions of GHRH signalling in sleep regulation and energy metabolism, suggesting that these neurons play broader roles that may shift away from growth control as GH secretion naturally declines in adulthood.

### 5.1 Sleep

GHRH plays a significant role in sleep regulation, particularly in the promotion of non–rapid eye movement sleep (NREMS). The largest pulse of GH typically occurs at sleep onset and coincides with the emergence of electroencephalogram slow‐wave activity,^9^ suggesting a functional link between GHRH signalling and sleep induction. Recordings in mice from GHRH neurons during sleep show a functional link between increased GHRH activity and NREMS.^6^ Rodent studies have shown that intracerebroventricular administration of GHRH rapidly enhances slow‐wave NREMS, likely through GHRHR activation in the preoptic area and cortex, which stimulates sleep‐promoting GABAergic neurons.^7^, ^102^ Supporting this, *Ghrhr* ^*lit/lit*^ mice exhibit reduced NREMS,^11^ highlighting the receptor's importance in sleep homeostasis. Similar effects have been observed in humans although the response to GHRH is diminished in older adults and in women,^103^ likely due to age‐ and sex‐related declines in HPS axis activity. Interestingly, GHRH promotes NREMS only when administered prior to natural sleep periods,^104^ suggesting that it maintains, rather than initiates, sleep. Moreover, these sleep‐promoting effects are independent of downstream HPS axis components, as neither GH/IGF1 administration, SST analogues, nor hypophysectomy alter NREMS.^105^, ^106^, ^107^ This indicates that feedback from the HPS axis may instead regulate the transition between sleep stages by modulating GHRH activity.

### 5.2 Glucose metabolism

Emerging evidence suggests that GHRH neurons are responsive to metabolic cues and may play a broader role in energy homeostasis than previously appreciated. A small subset (~1%) of GHRH neurons in the mouse ARH are polysynaptically connected to the pancreas^8^ forming a pathway that may contribute to the regulation of peripheral glucose metabolism. GHRH has been shown, primarily in ex vivo islet preparations, to directly stimulate insulin secretion from pancreatic β‐cells,^108^ indicating a functional link between hypothalamic GHRH signalling and pancreatic output. Further work in mice has shown that acute glucose deprivation, induced by agents such as 2‐deoxyglucose or insulin, activates GHRH neurons as indicated by c‐Fos expression.^8^ Chronic glucose deprivation alters GHRH neuron morphology, leading to a reduction in dendritic spines and an increase in SST appositions,^8^ suggesting an inhibition in response to metabolic stress. Mechanistically, GHRH neurons are depolarised by hypoglycaemia,^9^ a response that may be partially mediated by ghrelin, though studies using rat hypothalamic explants—devoid of circulating ghrelin—indicate that glucose directly inhibits GHRH release.^76^ This direct sensitivity is supported by the expression of glucokinase in mouse GHRH neurons,^9^ implicating a cell‐intrinsic glucose sensing mechanism that allows these neurons to respond dynamically to changes in systemic glucose levels.

## 6 FUTURE DIRECTIONS

The HPS axis represents a precisely regulated neuroendocrine feedback system, yet the mechanisms by which hypothalamic GHRH neurons generate and modulate GH pulses remain incompletely understood. GHRH neuron‐specific manipulations combined with repeated GH sampling and in vivo calcium or fibre‐photometry recordings (particularly across sleep–wake transitions) will be crucial to reveal the causal link between GHRH dynamics and GH output. Mechanistic insights about GHRH neurons typically derive from rodents, but new human hypothalamic resources—including single‐cell and spatial transcriptomic atlases such as the Human HYPOMAP^62^—now permit greater exploration of the molecular landscape involved with GHRH neuron activity. Mapping receptor and co‐transmitter expression through these atlases will provide a foundation for understanding how GHRH neurons might be different and differently modulated between species.

At the circuit level, the next challenge is to connect wider neural activity to neuroendocrine activity with temporal precision. pSTAT5 mapping identifies widespread GH‐responsive nodes, yet how these areas feedback to influence GHRH and SST populations across time and endocrine states remains unresolved. Integrating functional manipulations with spatial transcriptomics and viral connectomics will help define the broader network architecture including ARH microcircuits and feedback loops that shape pulsatile GH release. Ultimately, coupling neural readouts with real‐time endocrine signals will illuminate the conditional rules governing GH secretion and the state‐dependent logic of growth regulation.

## 7 CONCLUSIONS

Decoding how GHRH neurons orchestrate pulsatile GH secretion remains central to understanding growth. Emerging molecular, genetic and circuit‐level tools—together with new human‐based model systems—will enable detailed dissection of both the organisational and developmental trajectories of GHRH neurons and their broader networks governing growth control. Moreover, unravelling the molecular foundations of pulsatile GH release, particularly the contributions of synaptic plasticity, receptor dynamics and feedback integration, will deepen our understanding of growth regulation across physiological states. Ultimately, clarifying these circuits holds promise not only for advancing fundamental neuroendocrinology but also for identifying therapeutic targets for growth disorders, metabolic disease, and sleep‐related pathologies.

## AUTHOR CONTRIBUTIONS

**Bradley B. Jamieson:** Conceptualization; writing – original draft; writing – review and editing.

## CONFLICT OF INTEREST STATEMENT

The author declares no conflict of interest.

## Data Availability

Data sharing not applicable to this article as no datasets were generated or analysed during the current study.
